# Differences in the preferred food tastes characteristics in patients with depressive disorder and healthy subjects

**DOI:** 10.1192/j.eurpsy.2021.874

**Published:** 2021-08-13

**Authors:** A. Pluciute, V. Adomaitienė, V. Steibliene, L. Jarutiene, E. Bartkiene, V. Lele, D. Cernauskas, D. Klupsaite, D. Žadeikė, G. Juodeikiene

**Affiliations:** 1 Psychiatry, Lithuanian University of Health Sciences, Kaunas, Lithuania; 2 Food Safety And Quality, Lithuanian University of Health Sciences, Kaunas, Lithuania; 3 Food Science And Technology, Kaunas University of Technology, Kaunas, Lithuania

**Keywords:** depression, taste, food

## Abstract

**Introduction:**

There is no doubt that the symptoms of depression is the loss of appetite and loss the ability to taste food. However there is unanswered question how depression disorder impact different preferences of food tastes, which was sought to be explored in this study.

**Objectives:**

were to evaluate changes in characteristics of food tastes in patients with depressive disorder and healthy controls; and to find the association with clinical expression of depressive severity.

**Methods:**

74 patients with depressive disorder (according DSM-V, MINI 6.0.0) and 38 healthy controls, 18 to 55 age old, were included into this study. The subjects were interviewed using the sociodemographic and the food sensory questionnaires. The severity of depression was rated using Montgomery-Asberg Depression Rating Scale (MADRS).

**Results:**

There were significantly more patients with depressive disorder in comparison to healthy controls preferred non-spicy taste of food (66.2 % vs. 47.4 % respectively, p=0.025) and non-sour taste of food (66.2% vs 50.0 %, respectively, p=0.015), without significant differences in preference of salty and sweet food tastes. Among study patients with depressive disorder, the majority (71.6%) suffered from moderate severe depression, 23 % - severe depression and 5.4 % had mild severity depression. The preferences of tastes of the food (sour, sweet, salty, spicy) were independent of the severity of the depressive disorder (p>0.05)

**Conclusions:**

Patients with depressive disorder prefer non-spicy and non-sour food tastes, without differences in salty and sweet foods; it have found independent of the severity of the depressive disorder.

